# Random Phase Approximation Correlation Energy Using Real-Space Density Functional Perturbation Theory

**DOI:** 10.1021/acs.jctc.5c00528

**Published:** 2025-06-12

**Authors:** Boqin Zhang, Shikhar Shah, John E. Pask, Edmond Chow, Phanish Suryanarayana

**Affiliations:** † College of Engineering, 1372 Georgia Institute of Technology, Atlanta, Georgia 30332, United States; ‡ College of Computing, Georgia Institute of Technology, Atlanta, Georgia 30332, United States; § Physics Division, 4578 Lawrence Livermore National Laboratory, Livermore, California 94550, United States

## Abstract

We present a real-space method for computing the random phase approximation (RPA) correlation energy within Kohn–Sham density functional theory, leveraging the low-rank nature of the frequency-dependent density response operator. In particular, we employ a cubic-scaling formalism based on density functional perturbation theory that circumvents the calculation of the response function matrix, instead relying on the ability to compute its product with a vector through the solution of the associated Sternheimer linear systems. We develop a large-scale parallel implementation of this formalism using the subspace iteration method in conjunction with the spectral quadrature method while employing the Kronecker product-based method for the application of the Coulomb operator and the conjugate orthogonal conjugate gradient method for the solution of the linear systems. We demonstrate convergence with respect to key parameters and verify the method’s accuracy by comparing with plane-wave results. We show that the framework achieves good strong scaling to many thousands of processors, reducing the time to solution for a lithium hydride system with 128 electrons to around 150 s on 4608 processors.

## Graphical abstract

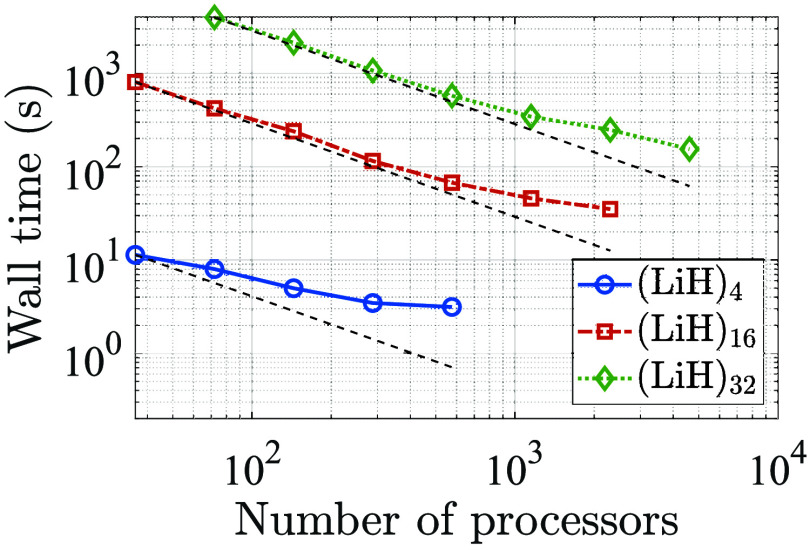

## Introduction

Over the past few decades, quantum mechanical calculations have become indispensable in materials and chemical sciences research, providing both fundamental insights and predictive power. Among the various first-principles approaches, Kohn–Sham density functional theory (DFT)^1,2^ has emerged as one of the most widely used, largely due to its versatility, relative simplicity, and favorable accuracy-to-cost ratio. Nevertheless, solving the Kohn–Sham equations remains computationally demanding, imposing significant limitations on the size and complexity of systems as well as the time scales that can be explored. These challenges become especially acute with the choice of more advanced exchange-correlation functionals.

The exchange-correlation functional in Kohn–Sham DFT is used to model both electron exchange, a quantum mechanical effect enforcing the Pauli exclusion principle, and electron correlation, which captures the dynamic interactions between electrons. In particular, exchange-correlation functionals can be classified by their accuracy and complexity within the conceptual framework of Jacob's Ladder,^3^ where each higher rung represents a more advanced and generally more accurate representation of exchange and correlation. The fifth and highest rung includes the random phase approximation (RPA) correlation energy, which incorporates many-body effects and can be derived from the adiabatic connection fluctuation dissipation (ACFD) theorem,^4^ ideally used alongside exact exchange. It can capture van der Waals interactions, eliminate self-interaction errors, and is applicable to both small-gap and metallic systems, enabling benchmark results for condensed matter systems.^5,6^ In particular, it has been found to offer better predictive capabilities than lower-rung functionals for a range of properties, including surface energy, adsorption energy, binding energy, cohesive energy, and lattice constants.^7–14^

The RPA correlation energy is expressed in terms of the Coulomb operator and the noninteracting Kohn–Sham density response function at imaginary frequency, which depends on both the occupied and unoccupied orbitals unlike lower-rung functionals that require only the occupied orbitals. Standard RPA correlation energy calculations^15–17^ exhibit quartic scaling with the number of grid points, and consequently with system size, while being associated with a very large computational prefactor. As a result, RPA calculations can be orders of magnitude more expensive than commonly used local/semilocal exchange-correlation functionals. This has motivated the development of approaches with reduced prefactor and/or scaling,^18–28^ as well as efficient/scalable parallel implementations.^26,27,29^ However, these frameworks are based on the plane-wave method,^30^ which confines calculations to periodic boundary conditions due to the underlying Fourier basis, necessitating artificial periodicity with large vacuum regions for finite systems such as molecules and clusters, as well as for semi-infinite systems like surfaces and nanotubes. Additionally, a neutralizing background density is required to prevent Coulomb divergences when treating charged systems, and the method’s reliance on fast Fourier transforms (FFTs) can hamper scalability on large-scale parallel computing platforms.

In view of the limitations of the plane-wave method, various approaches using systematically improvable, localized representations have been developed over the past two decades.^31–46^ Among these, finite-difference methods^47^ stand out as perhaps the most mature and widely used to date. By discretizing all relevant quantities on a real-space grid, these methods maximize computational locality while accommodating Dirichlet as well as periodic/Bloch-periodic boundary conditions and combinations thereof. This capability allows for the efficient and accurate treatment of finite, semi-infinite, and bulk 3D materials. Additionally, convergence is governed by a single parameter, i.e., grid spacing, and the method’s inherent simplicity and locality, along with its avoidance of communication-intensive transforms like FFTs, enable efficient scaling on large-scale parallel computing platforms. In particular, these methods can significantly outperform their plane-wave counterparts using local, semilocal, and hybrid exchange-correlation functionals, with increasing advantages as the number of processors is increased.^48–50^ Furthermore, they are capable of exploiting the decay of electronic interactions with distance, which has enabled the study of very large systems containing a million atoms for local/semilocal exchange-correlation.^51,52^ However, the RPA correlation energy has not been implemented within the real-space method heretofore, to the best of our knowledge, which provides the motivation for the present work.

In this work, we develop a real-space framework for calculating the RPA correlation energy within Kohn–Sham DFT, leveraging the low-rank nature of the frequency-dependent density response operator to avoid explicit construction of the full response matrix. In particular, we employ a density functional perturbation theory (DFPT)^53,54^ -based formalism for evaluation of the matrix vector products via the Sternheimer linear systems, reducing the overall scaling to cubic. We develop a highly scalable parallel implementation based on the subspace iteration^55^ and spectral quadrature (SQ)^56,57^ methods, while employing a Kronecker product-based scheme^50,58^ for application of the Coulomb operator and the conjugate orthogonal conjugate gradient (COCG) method^59,60^ for solution of the linear systems. We demonstrate convergence with respect to key parameters, verify the method’s accuracy by comparison with plane-wave results, and show that the framework achieves excellent strong scaling to many thousands of processors.

The remainder of this article is organized as follows. First, we discuss the DFPT-based approach for computing the RPA correlation energy. Next, we describe its implementation within the open-source SPARC electronic structure code^48,49^ and evaluate its accuracy and performance. Finally, we provide concluding remarks and outline potential directions for future work.

## Formulation

The RPA correlation energy can be written as^61^

1 $$E_{c}=\frac{1}{2\pi }\int_{0}^{\infty }\mathrm{Tr}\left[\log\left(I-\chi_{0}\left(i\omega \right)\nu \right)+\chi_{0}\left(i\omega \right)\nu \right]d\omega$$

where Tr[·] represents the trace operator, log denotes the natural logarithm, I is the identity operator, χ_0_(*i*ω) is the noninteracting Kohn–Sham density response function at imaginary frequency *i*ω, and ν is the Coulomb operator. Considering isolated systems or extended systems with Γ-point Brillouin zone integration, the response function for closed-shell systems when neglecting spin can be written as^62,63^

2 $$\chi_{0}\left(r,r^{\prime };i\omega \right)=2\sum_{j}\sum_{k}\frac{(f_{j}-f_{k})\psi_{j}\left(r\right)\psi_{k}\left(r\right)\psi_{k}\left(r^{\prime }\right)\psi_{j}\left(r^{\prime }\right)}{\varepsilon_{j}-\varepsilon_{k}-i\omega }$$

where ψ and ε are the eigenfunctions (orbitals) and eigenvalues of the Hamiltonian H :

3 $$H\psi_{n}=\varepsilon_{n}\psi_{n}$$

the sums over the indices *j* and *k* run over both the occupied and unoccupied orbitals, and *f* ∈ {0,1} are the occupations such that 2 ∑_*n*_ *f*_*n*_ = *N*_*e*_, *N*_*e*_ being the total number of electrons. In pseudopotential real-space density functional theory, the Hamiltonian takes the form:^44,64^

4 $$H≔-\frac{1}{2}\nabla^{2}+V_{\mathrm{xc}}+\phi +V_{\mathrm{nl}}$$

where ∇^2^ denotes the Laplacian, *V*_xc_ is the exchange-correlation potential, *V*_nl_ is the nonlocal pseudopotential operator, and ϕ is the electrostatic potential, which is the solution to the Poisson equation:

5 $$-\frac{1}{4\pi }\nabla^{2}\phi =\rho +b$$

with ρ and *b* being the electron and pseudocharge densities, respectively. Note that since χ_0_ is a negative definite operator, the RPA correlation energy is well defined in all instances.

The RPA correlation energy in eq 1 can be evaluated within the real-space method as

6 $$E_{c}\approx \frac{1}{2\pi }\int_{0}^{\infty }\sum_{n=1}^{N_{d}}\left(\log\left(1-\lambda_{n}\left(i\omega \right)\right)+\lambda_{n}\left(i\omega \right)\right)d\omega$$

where *N*_*d*_ is the number of grid points and λ_*n*_ are the eigenvalues of the χ_0_ ν-matrix, or equivalently those of the χ̃_0_ = ν^1/2^ χ_0_ ν^1/2^-matrix, which shares the same eigenvalues as χ_0_ ν, but is generally more efficient and convenient for use in computations. At any given frequency, the contribution to the correlation energy can be determined by first calculating the χ_0_-matrix, and then the χ̃_0_-matrix, followed by a computation of its eigenvalues. The calculation of the χ_0_-matrix using eq 2 scales as $O\left(N_{d}^{4}\right)$ with respect to the number of grid points, *N*_*d*_, while its computer storage scales quadratically, $O\left(N_{d}^{2}\right)$ , leading to the overall RPA correlation energy calculation having the same scaling behavior. Given that the number of grid points typically ranges from 400 to 30,000 per atom,^35^ the calculation of the RPA correlation energy is prohibitively expensive, restricting such simulations to particularly small systems. Furthermore, the calculation of the χ_0_-matrix requires unoccupied orbitals, which are not readily available from standard Kohn–Sham calculations.

In view of the above, it is preferable to use an iterative eigensolver that avoids the need to store the χ_0_ or χ̃_0_ matrices, requiring only the evaluation of matrix-vector products. Moreover, when computing such products, it is desirable to require only the occupied Kohn–Sham orbitals and eigenvalues. This can be accomplished within the framework of DFPT.^53,54^ In particular, treating the vector to be multiplied with the χ_0_-matrix as a perturbation in the potential Δ*V*, it follows from the definition of the density response function that

7 $$\chi_{0}\left(\Delta V\right)=\Delta \rho$$

where Δρ is the corresponding perturbation in the electron density. In so doing, the evaluation of the product of the χ_0_-matrix with the vector Δ*V* reduces to the calculation of the perturbation in the electron density Δρ due to the perturbation in the potential Δ*V*. The perturbation in the electron density Δρ can itself be written as

8 $$\Delta \rho =4\sum_{n=1}^{N_{s}}R\left[\psi_{n}\left(\Delta \psi_{n}\right)\right]$$

with Δ*ψ*_*n*_ being the perturbation in the orbitals and R representing the real part of the complex-valued quantity. The perturbation in the orbitals due to the perturbation in the potential Δ*V*, as presented in the literature,^18,23–25,65,66^ can be written as the solution to the linear Sternheimer equation:

9 $$\left(H-\varepsilon_{n}I-i\omega I\right)\left(\Delta \psi_{n}\right)=-\psi_{n}\left(\Delta V\right),,n=1,2,...N_{s}$$

where *N*_*s*_ is the number of occupied states. Given that the Hamiltonian H is symmetric, $H-\varepsilon_{n}I-i\omega I$ is complex symmetric. In particular, $H-\varepsilon_{n}I-i\omega I$ has the same eigenvectors as H , with the eigenvalues shifted by *ε*_*n*_ along the negative real-axis and ω along the negative imaginary axis. Since *ε*_*n*_ are the eigenvalues of H , the linear systems become increasingly ill-conditioned and therefore more difficult to solve as ω → 0 and as the eigenvalues become more clustered near *ε*_*n*_. Additionally, the systems become harder to solve as the operator becomes more indefinite, a behavior that is more pronounced for states near the Fermi level. To enable the use of efficient subspace-based iterative eigensolvers without being constrained by storage limitations, we assume a low-rank decomposition of the χ̃_0_-matrix,^18,23–25^ whereby the RPA correlation energy is approximated as

10 $$E_{c}\approx \frac{1}{2\pi }\int_{0}^{\infty }\sum_{n=1}^{N_{r}}\left(\log\left(1-\lambda_{n}\left(i\omega \right)\right)+\lambda_{n}\left(i\omega \right)\right)d\omega$$

with *N*_r_ being the rank of the decomposition. Indeed, the eigenvalues of the χ̃_0_-matrix rapidly decay to zero, as shown in Figure 1. Furthermore, under the transformation *g*(*x*) = log­(1 – *x*) + *x*, eigenvalues near zero become closer to zero, whereby their contribution to the RPA correlation energy is diminished. Also, the lower frequencies contribute more significantly to the correlation energy.

**Figure 1:**
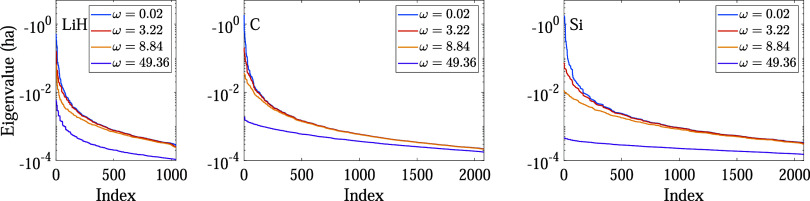
Decay in the eigenvalues of the χ̃_0_-matrix for 2-atom cells of lithium hydride (left), carbon (middle), and silicon (right), Γ-point Brillouin zone integration, and Perdew–Burke–Ernzerhof (PBE) exchange-correlation functional.

In summary, while calculating the eigenvalues of interest, i.e., the lowest *N*_r_ eigenvalues of χ̃_0_, the product of the χ_0_-matrix with any Δ*V* vector can be calculated by solving the Sternheimer equations for the different Δψ vectors, which are then used to calculate the Δρ vector. In this way, the calculation of the unoccupied states is avoided, and the formalism has a reduced scaling of $O\left(N_{r}N_{s}N_{d}\right)∼O\left(N_{d}^{3}\right)$ , while being accompanied by a relatively small prefactor. Indeed, the prefactor increases for systems that require a larger decomposition rank *N*_r_, and in cases with a high density of states near the Fermi level, such as in metallic systems, where the Sternheimer equations become more challenging to solve. It is worth noting that by choosing the standard basis vectors for Δ*V*, the DFPT-based formalism can also be used to calculate the χ_0_-matrix with $O\left(N_{s}N_{d}^{2}\right)∼O\left(N_{d}^{3}\right)$ scaling, rendering the overall scaling to also be $O\left(N_{d}^{3}\right)$ , although this approach still requires storing the complete χ_0_-matrix and has a substantially larger prefactor.

In the case of non-Γ-point Brillouin zone integration, the RPA correlation energy in eq 1 involves an integration over the Brillouin zone with the wavevector-dependent response function at each wavevector also requiring a Brillouin zone integration. The above DFPT-based formalism can be extended to such cases by solving two Sternheimer equations to compute the perturbation in the electron density: one for the perturbation in the orbitals and another for the perturbation in their complex conjugates. Although this increases the prefactor, the $O\left(N_{d}^{3}\right)$ scaling is retained for a fixed number of Brillouin zone wavevectors. To account for fractional occupations, as encountered in metallic systems, the perturbation in the occupations must also be included in the calculation of the electron density perturbation.^67,68^ This only marginally increases the cost of the calculations, while retaining the $O\left(N_{d}^{3}\right)$ scaling.

## Implementation

We now discuss the implementation of the DFPT-based formalism for the calculation of the RPA correlation energy within the SPARC^48,49^ electronic structure code. SPARC is based on the real-space finite-difference method, wherein high-order centered finite differences are used to approximate derivative operators and the trapezoidal rule is applied for spatial integrations. It utilizes norm-conserving pseudopotentials and a local formulation for the electrostatics. The sparse Hamiltonian matrix is not explicitly computed or stored; instead, it is applied as an operator using a matrix-free scheme. In this work, we focus on the non-self-consistent RPA correlation energy, which is calculated at the electronic ground state of a lower-rung exchange-correlation functional. The ground state orbitals and eigenvalues, computed using SPARC, are stored in files and subsequently read during calculation of the correlation energy.

The pseudocode for the RPA correlation energy calculation is presented in Algorithm 1, where the scaling of the key computational operations is also been listed. The integral over the frequency is approximated using the Gauss-Legendre quadrature. At each frequency in the quadrature rule, the contribution to the RPA correlation energy is determined by using the subspace iteration method,^55^ a generalization of the power method, in conjunction with the Gauss Spectral Quadrature (SQ) method.^56,57^ The loop over the frequency proceeds from highest to lowest since the solution of the Sternheimer equations becomes more challenging at the lower frequencies, given that the coefficient matrix becomes more ill-conditioned as ω → 0. In particular, starting with higher frequencies allows the eigenvector subspace of χ̃_0_ computed at a given frequency to serve as a good initial guess for the subsequent lower frequency, reducing the number of iterations in the subspace iteration method and, therefore, the overall computational cost. At each frequency, the convergence of the associated RPA correlation energy is used as the stopping criterion for the subspace iteration method.

In the subspace iteration method, the χ̃_0_-matrix is multiplied by trial vectors that, upon convergence, span the eigenspace corresponding to the lowest *N*_*r*_ eigenvalues. For numerical stability, the trial vectors are orthogonalized in each iteration using the Cholesky factor of the overlap matrix, ensuring that the vectors do not become linearly dependent, an issue that arises due to the tendency of a power-like method to converge to the dominant eigenvalue. Note that in recent work,^60^ the subspace iteration method was used with second-degree polynomial filtering, requiring three χ̃_0_-matrix products per iteration, whereas the current methodology requires only a single χ̃_0_-matrix product per iteration, making the current implementation significantly faster. The RPA correlation energy at the given frequency is calculated using the Gauss SQ method applied to the χ̃_0_-matrix projected into the subspace of the trial vectors while not employing any truncation of the off-diagonal components. In particular, starting with each of the standard basis vectors in the subspace spanned by the trial vectors, the Lanczos iteration is used to generate the corresponding tridiagonal matrix. The eigenvalues and square of the first components of the eigenvectors of this matrix provide the nodes and weights for the quadrature rule. The choice of Gauss SQ rather than subspace diagonalization is motivated by its highly scalable nature.^69,70^ In addition, the prefactor associated with the method is expected to be relatively small due to the nature of the function being integrated, namely, *g*(*x*) = log­(1 – *x*) + *x*, for which quadrature orders as low as 3 are sufficiently accurate in practice.

The multiplication of the χ̃_0_-matrix with any trial vector proceeds as follows. First, the product of the ν^1/2^-matrix with the vector is calculated using the real-space Kronecker product-based formalism,^50,58^ which has comparable efficiency to the fast Fourier transform (FFT) method, without the restriction of periodic boundary conditions. Next, the product of the χ_0_-matrix with the resulting vector is evaluated using the DFPT formalism described in the previous section. In particular, the Sternheimer equation is solved using the conjugate orthogonal conjugate gradient (COCG) method,^59,60^ which represents a generalization of the conjugate gradient (CG) method to complex-symmetric linear systems. The initial guess for the linear solver is constructed using Galerkin projection, where the components corresponding to the occupied Kohn–Sham states are removed from the initial residual.^60^ Note that though the solution of the linear system corresponding to the previous larger frequency likely serves as a good initial guess for the subsequent lower frequency, this results in a significant increase in the computer memory required; hence, the strategy is not adopted here. Also note that due to load imbalance issues in parallel computations, i.e., the Sternheimer linear system solution is more challenging for eigenvectors corresponding to lower eigenvalues of the χ̃_0_-matrix, the trial vectors were cyclically reordered by multiplying with a permutation matrix in recent work.^60^ However, we found that this strategy does not provide any gains here, as the trial vectors are not the eigenvectors themselves but merely span the same subspace, a consequence of using the SQ method rather than an eigensolver for the subspace eigenproblem.

The implementation applies different parallelization strategies to each step, depending on the operations to be carried out in that step. In particular, when evaluating the χ̃_0_-matrix product with the trial vectors, the multiplication of the ν^1/2^ matrix with the vectors uses a one-level parallelization over the different vectors, while the χ_0_-matrix product with the vectors employs a two-level parallelization, first over the trial vectors and then over the Sternheimer linear systems. After evaluating the matrix-vector products, both the resulting matrix and the trial vector matrix are redistributed into block-cyclic form, with their multiplication performed using ScaLAPACK^71^ routines on the subset of processors over which the trial vectors are parallelized. The resulting subspace matrix is then redistributed across a two-level group of processors, with each subgroup storing the matrix partitioned row-wise among the processors within that subgroup. The Gauss SQ implementation uses two levels of parallelization: first, across the different standard basis vectors within the processor group, and second, over the matrix-vector multiplications involved in the Lanczos iteration within the processor subgroup. After the SQ process, the orthogonalization of the χ̃_0_-matrix multiplied vectors, i.e., calculation of the overlap matrix, Cholesky factorization, and subspace rotation, are all performed using ScaLAPACK routines. The layout of the trial vectors is then restored to that used for the product of the χ̃_0_-matrix with the trial vectors.

The key computational step in the above methodology is the solution of the Sternheimer linear systems, the cost of which scales as $O\left(N_{d}\right)$ each. Since the product of the χ_0_-matrix with each vector involves *N*_*s*_ such linear systems, and assuming the number of iterations in the subspace iteration method is independent of the system size, the total number of matrix-vector products is $O\left(N_{r}\right)$ , leading to an overall scaling of $O\left(N_{r}N_{s}N_{d}\right)∼O\left(N_{d}^{3}\right)$ . Indeed, the generation of the initial guess for each linear system scales as $O\left(N_{s}N_{d}\right)$ , which leads to an overall scaling of $O\left(N_{r}N_{s}^{2}N_{d}\right)∼O\left(N_{d}^{4}\right)$ . However, for small to moderate system sizes, this cost constitutes only a small fraction of the total cost, making the implementation effectively cubic scaling in practice. In terms of computer memory, the primary storage requirements arise from the trial vectors, which scale as $O\left(N_{r}N_{d}\right)$ , and the Kohn–Sham orbitals, which scale as $O\left(N_{s}N_{d}\right)$ . This results in an overall memory scaling of $O\left(N_{r}N_{d}+N_{s}N_{d}\right)∼O\left(N_{d}^{2}\right)$ . Although this scaling is the same as that of storing the χ̃_0_-matrix in the direct approach for computing the RPA correlation energy, the associated prefactor is substantially smaller. It is important to note that although *N*_*d*_ is significantly larger than *N*_*s*_, the memory per processor is primarily determined by the need to store all orbitals on each processor for the initial guess calculation. This is because parallelization is performed over the trial vectors, effectively distributing their storage across processors, while the orbitals must be fully retained on each processor. To circumvent the need for the quartic scaling initial guess calculation and associated storage requirements, we have also implemented Laplacian-based preconditioning based on the Kronecker product formalism^50^ and the block variant of the COCG linear solver.^60^ Indeed, preliminary results indicate that these modifications yield performance comparable to the current framework for small- to moderate-sized systems, while preserving the overall cubic scaling, as the generation of the initial guess can be omitted due to its marginal effect in this setting.^72^ 
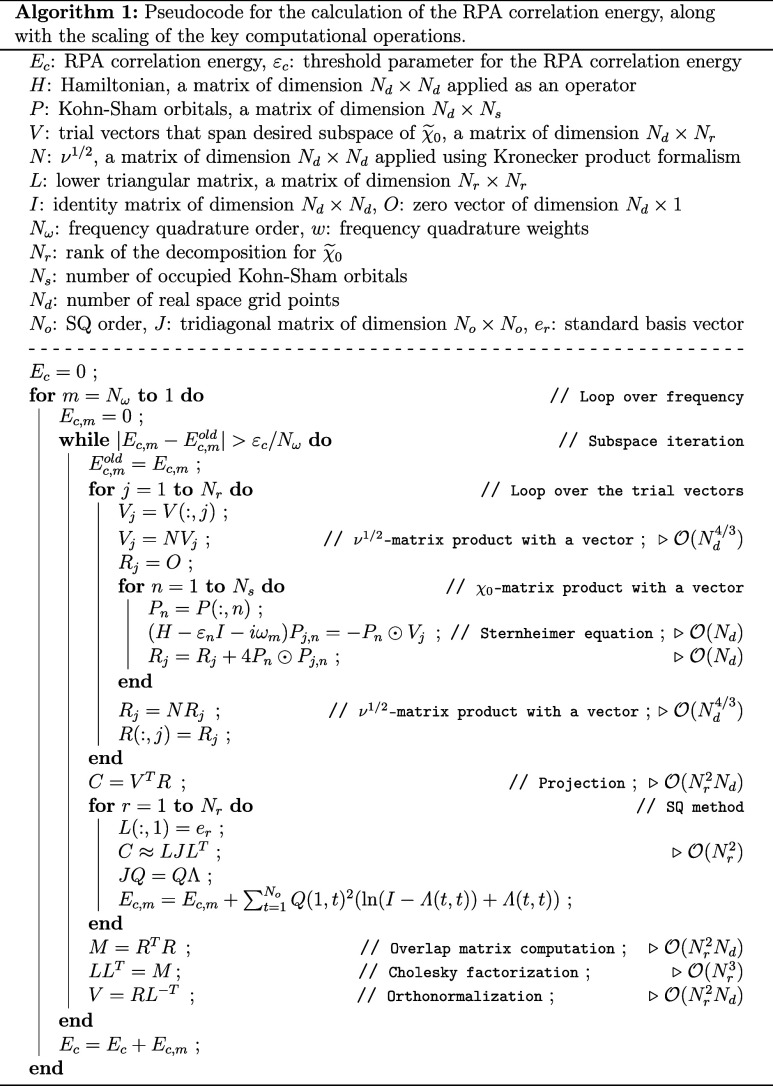


## Results and Discussion

We now study the accuracy and performance of the DFPT-based framework developed in this work for the calculation of the RPA correlation energy. We consider cells of silicon (Si), carbon (C), and lithium hydride (LiH), with the atoms randomly perturbed in the strong scaling test using the Γ-point for Brillouin zone integration. In all simulations, including electronic ground state calculations, we use the Perdew–Burke–Ernzerhof (PBE) exchange-correlation functional^73^ and ONCV pseudopotentials^74^ with nonlinear core corrections from the SPMS table,^75^ which have 4, 4, 3, and 1 electrons in valence for Si, C, Li, and H, respectively. In all instances, we set the order of the Gauss-Legendre quadrature for the integral over the frequency as *N*_ω_ = 8.

### Convergence with respect to parameters

We first study the convergence of the RPA correlation energy with respect to the key parameters: normalized rank of the decomposition *N*_*r*_/*N*_*e*_, where *N*_*e*_ is the total number of electrons; tolerance *ε*_*s*_ for solving the Sternheimer equation, prescribed on the relative residual; real-space grid spacing *h*; and SQ order *N*_*o*_. We consider 8-atom cubic cells with dimensions of 10.3, 6.7, and 7.6 bohr for the Si, C, and LiH systems, respectively, containing 32, 32, and 16 electrons. Unless specified otherwise, we employ *N*_*o*_ = 7, *ε*_*s*_ = 10^–3^, *N*_*r*_/*N*_*e*_ = 260, subspace iteration RPA correlation energy threshold of *ε*_*c*_ = 10^–6^ ha/atom, and *h* = 0.20, 0.15, and 0.15 bohr for the Si, C, and LiH systems, respectively, resulting in *N*_*d*_ = 140,608, 91,125, and 132,651 grid points.

In Figure 2a, we plot the variation of the RPA correlation energy with respect to the normalized decomposition rank *N*_*r*_/*N*_*e*_. The error is defined with respect to the values obtained for *N*_*r*_/*N*_*e*_ = 350. We observe rapid convergence in the RPA correlation energy, with *N*_*r*_/*N*_*e*_ ∼70, 70, and 30 being sufficient to achieve chemical accuracy of ∼0.001 ha/atom for the Si, C, and LiH systems, respectively. The corresponding numbers for an accuracy of 1 × 10^–4^ ha/atom in the RPA correlation energy are ∼260, 260, and 170, respectively. These results confirm that the low-rank assumption of the χ̃_0_-matrix is a good approximation for the calculation of the RPA correlation energy.

**Figure 2:**
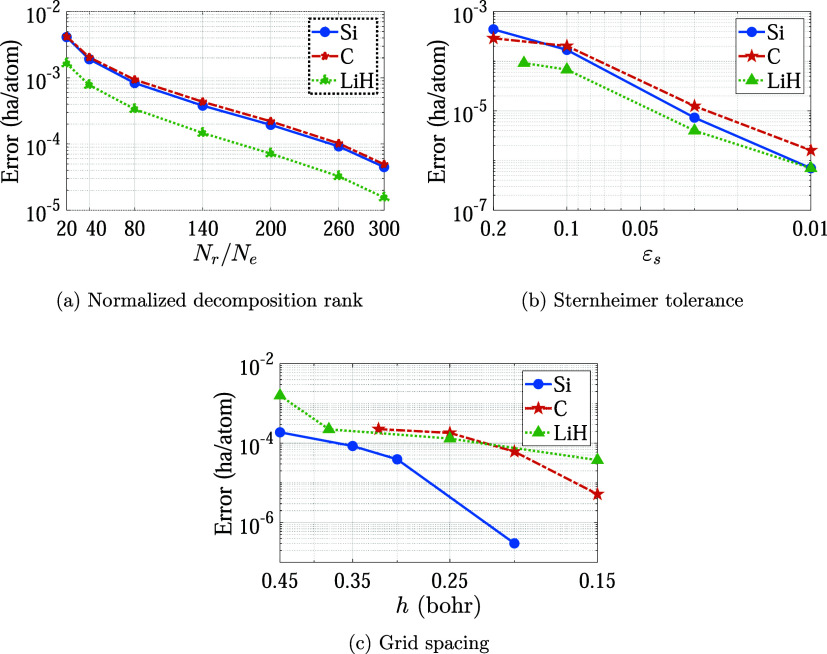
Variation of the RPA correlation energy with key parameters in the developed framework.

In Figure 2b, we plot the variation of the RPA correlation energy with respect to Sternheimer tolerance *ε*_*s*_. The error is defined with respect to the values obtained for *ε*_*s*_ = 10^–3^. We observe that there is rapid convergence, with even a loose tolerance of *ε*_*s*_ ∼0.2 sufficient to obtain a chemical accuracy of ∼0.001 ha/atom in the correlation energy. Notably, in recent work employing subspace diagonalization,^60^ the value of *N*_*r*_/*N*_*e*_ constrained how loosely the tolerance *ε*_*s*_ could be set, as the guess eigenvectors tended to become linearly dependent during the polynomial-filtered subspace iteration. However, this issue is not encountered in the current framework using SQ, which permits looser Sternheimer tolerances and therefore leads to significant speedups of up to a factor of 2 for the chosen systems.

In Figure 2c, we plot the variation of the RPA correlation energy with real-space grid spacing *h*. The error is defined with respect to the values corresponding to *h* = 0.15, 0.10, and 0.10 for the Si, C, and LiH systems, respectively. We observe that there is a rapid convergence in the energy. In particular, the correlation energy converges to within 10^–4^ ha/atom as the grid spacing is refined, achieving chemical accuracy of ∼0.001 ha/atom with a grid spacing of *h* ∼0.45 bohr. Notably, a similar grid spacing is required to achieve chemical accuracy for other choices of exchange-correlation functionals, confirming that an unnecessarily fine grid is not needed for convergence of the RPA correlation energy.

In Table 1, we present the variation of the RPA correlation energy with SQ order *N*_*o*_. We observe that there is rapid convergence in the energy. In particular, *N*_*o*_ = 4 is sufficient to achieve an accuracy of 10^–7^ ha/atom for each of the systems. This can be attributed to the very small spectral width of the χ̃_0_-matrix when projected onto the subspace of the trial vectors, e.g., O(1) ha for the systems studied here, and the relatively smooth nature of the function *g*(*x*) = log­(1 – *x*) + *x* for which SQ is being performed.^56^

**Table 1:** Variation of the RPA Correlation Energy with the SQ Order *N_o_*

*N_o_*	2	3	4
Si	–0.2091340	–0.2091346	–0.2091347
C	–0.2468244	–0.2468245	–0.2468246
LiH	–0.0790038	–0.0790044	–0.0790045

The selection of the parameters that are new to the developed formalism, i.e., normalized decomposition rank *N*_*r*_/*N*_*e*_, Sternheimer tolerance *ε*_*s*_, and SQ order *N*_*o*_, merits further discussion. Suitable values for *N*_*r*_/*N*_*e*_ and *ε*_*s*_ can be determined by assessing convergence with respect to these parameters, typically using a smaller representative system to minimize computational cost. Indeed, a Sternheimer tolerance of *ε*_*s*_ = 0.001 can generally be used to obtain chemical accuracy, although choosing a less strict value that achieves the desired accuracy can significantly accelerate the calculations. Since the function *g*(*x*) = log­(1 – *x*) + *x* remains fixed across systems, the SQ order *N*_*o*_ = 4 can generally be used in all calculations. While *N*_*o*_ = 3 is likely sufficient for achieving the desired accuracy, adopting *N*_*o*_ = 4 is recommended, as SQ constitutes only a small fraction of the total computational cost.

The above results pertain to insulating systems with Γ-point Brillouin zone integration. Initial findings suggest that the parameter choices remain similar when a full Brillouin zone integration is employed. In the case of metallic systems, while the quadrature order *N*_*o*_ is expected to remain unchanged, the values for the normalized decomposition rank *N*_*r*_/*N*_*e*_ and Sternheimer tolerance *ε*_*s*_ warrant further investigation.

### Accuracy

We next verify the accuracy of the developed framework through comparisons with the established plane-wave code ABINIT,^15^ which uses the direct approach for the calculation of the RPA correlation energy, i.e., construction of the *ν χ*_0_ matrix, followed by an eigendecomposition. In particular, we consider a 2-atom cubic cell with dimensions of 5.14, 3.36, and 4.38 bohr for the Si, C, and LiH systems, respectively, containing 8, 8, and 4 electrons. In SPARC, we employ a grid spacing of 0.20, 0.15, and 0.15 bohr for the Si, C, and LiH systems, respectively, resulting in *N*_*d*_ = 17,576, 12,167, and 27,000 grid points. In addition, we employ normalized decomposition ranks of *N*_*r*_/*N*_*e*_ = 350, Sternheimer tolerance of *ε*_*s*_ = 0.01, SQ order of *N*_*o*_ = 4, and subspace iteration correlation energy threshold of *ε*_*c*_ = 10^–5^ ha/atom. With these choices, the RPA correlation energies calculated by SPARC are converged to ∼10^–4^ ha/atom. In ABINIT, we used all unoccupied orbitals for the construction of the χ_0_-matrix. In addition, we employ plane-wave cutoffs of 135, 195, and 165 ha for the Si, C, and LiH systems, respectively. With these choices, the RPA correlation energies calculated by ABINIT are converged to ∼10^–4^ ha/atom.

In Table 2, we compare the RPA correlation energies computed by SPARC and ABINIT. We observe that there is very good agreement with differences of ∼10^–4^ ha/atom, which are well within the desired chemical accuracy of ∼0.001 ha/atom. Indeed, the agreement is expected to further increase on choosing larger plane-wave cutoffs in ABINIT; however, such simulations fail to execute on our computer cluster. Note that, based on the model developed in SPARC for the equivalent grid spacing in real-space calculations and the plane-wave cutoff in plane-wave calculations for the electronic ground state, the RPA correlation energy appears to converge faster in SPARC compared to ABINIT. This is likely due to the lower accuracy of the unoccupied states computed in ABINIT, as observed in the outputs. Also note that the current implementation of the RPA correlation energy is applicable to study molecules/clusters. However, we have not done so here because comparisons with plane-wave codes become even more challenging due to differences in boundary conditions and the large vacuum required, which significantly increases the degrees of freedom and prevents reaching the plane-wave cutoffs needed for a careful comparison.

**Table 2:** RPA Correlation Energy Computed by SPARC and ABINIT

	ABINIT (ha/atom)	SPARC (ha/atom)	difference (ha/atom)
Si	–0.20246	–0.20235	0.9 × 10^–4^
C	–0.21899	–0.21916	1.7 × 10^–4^
LiH	–0.06300	–0.06331	3.1 × 10^–4^

### Performance

We now study the performance of the developed framework for the calculation of the RPA correlation energy. In particular, we perform a strong scaling test, i.e., we study the variation time to solution as the number of processors is increased while holding the system size fixed. We consider 8-, 32-, and 64-atom cuboidal cells of LiH with dimensions of 7.6 × 7.6 × 7.6, 15.2 × 15.2 × 7.6, and 15.2 × 15.2 × 15.2 bohr, respectively, containing 16, 64, and 128 electrons. The processor count is varied from 36 to 576 for (LiH)_4_, from 36 to 2304 for (LiH)_16_, and from 72 to 4608 for (LiH)_32_. We employ a grid spacing of 0.40 bohr, resulting in *N*_*d*_ = 6,859, 27,436, and 54,872 grid points for (LiH)_4_, (LiH)_16_, and (LiH)_32_, respectively. In addition, we employ a normalized decomposition rank of *N*_*r*_/*N*_*e*_ = 36, Sternheimer tolerance of *ε*_*s*_ = 0.1, SQ order of *N*_*o*_ = 3, and subspace iteration energy threshold of *ε*_*c*_ = 5 × 10^–4^ ha/atom. With these choices, the RPA correlation energy is converged to within a chemical accuracy of ∼0.001 ha/atom. The simulations are performed on the Phoenix supercomputer at Georgia Institute of Technology, where each node has Dual Intel Xeon Gold 6226 CPUs @ 2.7 GHz (24 cores/node), DDR4-2933 MHz DRAM, and Infiniband 100HDR interconnect. The (LiH)_4_ system requires approximately 50 MB of RAM per processor for the processor counts considered. For the (LiH)_16_ system, the memory usage decreases from approximately 200 MB per processor on 36 processors to about 50 MB per processor on 2304 processors. Similarly, for the (LiH)_32_ system, the memory decreases from around 500 MB per processor on 72 processors to approximately 90 MB per processor on 4608 processors.

We present the parallel strong scaling results obtained in Figure 3. We observe that SPARC exhibits good scalability to thousands of processors, achieving efficiencies of 23, 36, and 40% for the (LiH)_4_, (LiH)_16_, and (LiH)_32_ systems, respectively, on the largest processor counts of 576, 2304, and 4608. Indeed, higher efficiencies are expected due to the inherently parallel nature of solving the Sternheimer linear systems, which employ a two-level parallelization over both trial vectors and Kohn–Sham orbitals. However, as the number of processors is increased, the solution of the Sternheimer system becomes less dominant, and the scaling becomes limited by the other key steps, including projection of the χ̃_0_-matrix onto the subspace spanned by the trial vectors and orthonormalization of the trial vectors. This is evident from Figure 4, where the breakdown of the timings in the strong scaling study has been presented. In particular, on the smallest number of processors, the solution of the Sternheimer equations takes 83, 95, and 96% of the total time, while on the largest number of processors, it occupies only 24, 63, and 82% of the total time. In particular, the projection and orthogonormalization steps scale relatively poorly, which significantly reduces the overall scaling efficiency. Indeed, the time taken by the Kronecker product scheme and SQ are relatively minor fractions of the total time. Note that even within the time spent solving the Sternheimer equations, the generation of the initial guess—computed simultaneously for all right-hand side vectors local to a processor—exhibits poor strong scaling, as the BLAS3 operations effectively reduce to BLAS2 operations with increasing processor count. Indeed, excluding the time spent on generating the initial guess, the efficiency of solving the Sternheimer linear systems on the largest number of processors is 82.4, 86.5, and 86.4% for the (LiH)_4_, (LiH)_16_, and (LiH)_32_ systems, respectively, with the loss in efficiency attributable to the load imbalance arising from the varying difficulty of the individual linear systems. We also observe from these results, based on the CPU times for the LiH systems at the smallest number of processors, i.e., 0.16, 9.56, and 85.70 CPU-hours for the (LiH)_4_, (LiH)_16_, and (LiH)_32_ systems, respectively, that the formalism scales nearly perfectly with the system size, i.e., $O\left(N_{d}^{3}\right)$ . Indeed, the cost is dominated by the solution of the Sternheimer linear systems, with the number of iterations required for their solution being independent of the system size, as shown in Table 3. The results also indicate that the number of iterations tends to increase for smaller values of ω and that there is relatively large variation in the number of iterations required for the different linear systems at each ω, as previously discussed.

**Figure 3:**
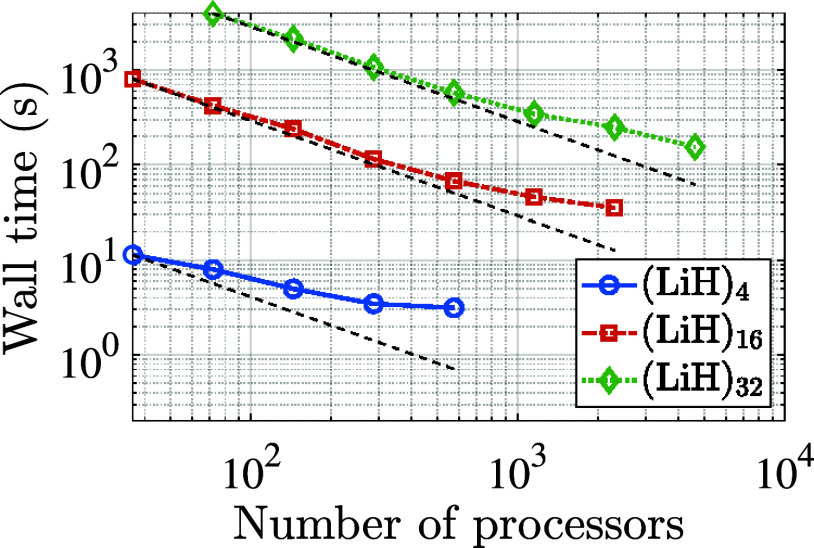
Strong scaling of SPARC for the calculation of the RPA correlation energy, where the dotted lines represent the ideal scaling.

**Figure 4:**
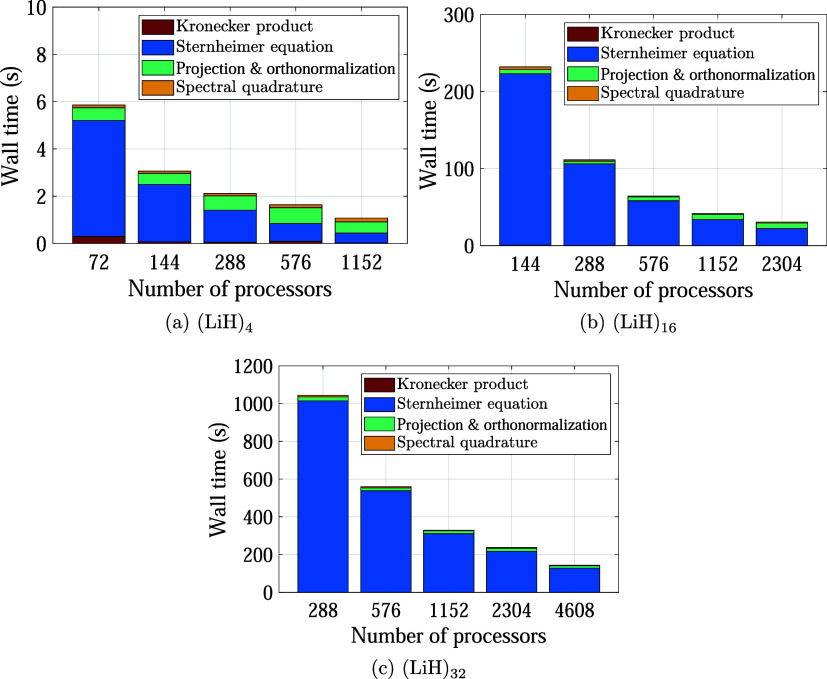
Breakdown of the timings in the strong scaling study (Figure 3) for LiH systems.

**Table 3:** Number of Iterations Required to Solve the Sternheimer Linear Systems in the Strong Scaling Study (Figure 3)

	mean			std. dev.			(min, max)		
ω	(LiH)_4_	(LiH)_16_	(LiH)_32_	(LiH)_4_	(LiH)_16_	(LiH)_32_	(LiH)_4_	(LiH)_16_	(LiH)_32_
49.37	2.0	2.0	2.0	0.0	0.0	0.0	(1, 2)	(1, 2)	(1, 2)
8.84	4.6	4.6	4.6	0.5	0.5	0.5	(3, 5)	(2, 5)	(2, 5)
3.22	6.7	6.6	6.7	1.5	1.6	1.6	(3, 9)	(2, 9)	(2, 9)
1.45	8.0	8.0	8.0	3.0	3.1	3.1	(4, 14)	(2, 14)	(2, 13)
0.69	8.3	8.2	8.1	4.0	4.0	4.1	(3, 20)	(2, 18)	(2, 18)
0.31	8.1	7.9	7.9	4.6	4.5	4.5	(4, 26)	(1, 25)	(0, 25)
0.11	7.9	7.7	7.7	4.8	4.8	4.5	(4, 33)	(0, 29)	(0, 30)
0.02	7.7	7.6	7.5	4.5	4.5	4.4	(4, 35)	(4, 30)	(0, 34)

In terms of comparison of the performance with the plane-wave code ABINIT, which uses the direct approach for the calculation of the RPA correlation energy, we consider the (LiH)_4_ system. We use a plane-wave cutoff of 75 ha, which converges the RPA correlation energy to ∼0.001 ha/atom, comparable to the accuracy of the SPARC results. The CPU time taken by ABINIT, which includes the electronic ground state calculation, is 34 h, while the corresponding time taken by SPARC is 0.75 h, achieving more than an order-of-magnitude speedup. Given SPARC’s cubic-scaling formalism compared to ABINIT’s quartic scaling, along with the greater parallel scalability of the DFPT-based approach, its advantages are expected to become more pronounced with increasing system size and the availability of more processors. This is expected even in the case of fractional occupations, i.e., metallic systems and/or non-Γ-point Brillouin zone integration, as discussed previously. It is worth noting that the developed framework is expected to be highly competitive with previous related approaches that rely on a low-rank approximation of the χ̃_0_-matrix,^18,23–25^ with increasing advantages as the system size grows. In particular, these earlier methods exhibit quartic scaling due to the use of an occupied-space projector in the matrix-vector products in the solution of the Sternheimer equations, a step that is bypassed in the present work through the use of a suitably generated initial guess and the COCG method. Compared to the cubic-scaling method of Kaltak et al.,^22^ the developed framework is expected to offer advantages when finer grid spacings are used, as it scales linearly with the number of grid points for a given system size, in contrast to the cubic scaling of Kaltak et al.^22^ Additional gains are anticipated when the number of electrons per atom and/or the rank of decomposition required by the present formalism are relatively low. Notably, the prefactor associated with the present framework can likely be reduced by employing interpolative separable density fitting (ISDF), a strategy utilized in the cubic-scaling formalism of Lu and Thicke,^28^ in conjunction with polynomial interpolation, as demonstrated in the DFPT context for phonons.^76^

The system sizes chosen for the strong scaling study above are relatively small with the largest system, (LiH)_32_, containing 128 electrons. These sizes were selected to ensure lowest time to solution of a few minutes on the available computational resources, which comprised a few thousand processors. Since the framework is not memory-limited, larger systems can be simulated with modest computational resources. For example, using the same parameters as the other LiH systems, the (LiH)_128_ system, which contains 512 electrons, can be simulated on 576 processors in ∼12.6 h, consistent with the framework’s cubic scaling. This demonstrates that systems with O(500−1000) electrons are routinely accessible to the developed framework.

## Concluding Remarks

In this work, we presented a real-space method for computing the RPA correlation energy within Kohn–Sham DFT, leveraging the low-rank nature of the frequency-dependent density response operator. In particular, we have employed a cubic-scaling formalism based on DFPT that circumvents the explicit construction of the response function matrix instead relying on the ability to calculate its product with a vector by solving the associated Sternheimer linear systems. We have developed a large-scale parallel implementation of this approach using the subspace iteration method in conjunction with the SQ method, while employing the Kronecker product-based formalism for the application of the Coulomb operator and the COCG method for the solution of the linear systems. We have demonstrated the convergence with respect to key parameters and verified the methods accuracy by comparing with plane-wave results. In addition, we have demonstrated that the framework exhibits good strong scaling to many thousands of processors, reducing the time to solution for a lithium hydride system with 128 electrons to around 150 s on 4068 processors.

The acceleration of the key computational kernels on GPUs, as implemented for local/semilocal^77^ and hybrid^58^ functionals, is expected to significantly reduce the time to solution, making it a promising avenue for future research. In particular, leveraging GPU acceleration for solving the Sternheimer linear systems is expected to result in substantial computational speedup. Other worthwhile directions include extending the framework to incorporate Brillouin zone integration, which requires solving two Sternheimer linear systems for each perturbation in every orbital and incorporating exact exchange within the DFPT-based formalism. Additionally, developing a self-consistent formulation and a large-scale parallel implementation for the RPA correlation energy represents a valuable avenue for further research.
